# Incidence and risk factors for postoperative myocardial infarction following hip and knee arthroplasty

**DOI:** 10.1302/2633-1462.78.BJO-2026-0065.R1

**Published:** 2026-08-11

**Authors:** Ahmed Al-Saadawi, Saran Singh Gill, Sara Sousi, Gareth G. Jones, Alex Bottle, Justin P. Cobb, Omar Musbahi

**Affiliations:** 1 Lister Hospital, East and North Hertfordshire Teaching NHS Trust, Stevenage, UK; 2 MSk Lab, Sir Michael Uren Hub, White City Campus, Imperial College London, London, UK; 3 School of Public Health, White City Campus, Imperial College London, London, UK

**Keywords:** Myocardial infarction, Hip arthroplasty, Knee arthroplasty, knee arthroplasty procedures, hip and knee arthroplasty, unicompartmental knee arthroplasty (UKA), total knee arthroplasty (TKA), joint arthroplasties, total hip arthroplasty (THA), logistic regression analysis, hip resurfacing arthroplasty, propensity scores

## Abstract

**Aims:**

Hip and knee arthroplasty are among the most commonly performed surgical procedures worldwide but carry a risk of cardiovascular complications, including myocardial infarction (MI). Therefore, this study aimed to assess the long-term incidence of MI following hip and knee arthroplasty, and subsequent mortality, in a UK-based cohort.

**Methods:**

A retrospective cohort study was conducted using linked Hospital Episode Statistics (HES), Office for National Statistics (ONS) mortality data, and Clinical Practice Research Datalink (CPRD) records. Patients undergoing total hip arthroplasty (THA), hip resurfacing arthroplasty (HRA), total knee arthroplasty (TKA), or unicompartmental knee arthroplasty (UKA) between 2 January 1998 and 29 March 2021 were identified. An inverse probability weighting approach based on propensity scores was used, and outcomes were assessed using weighted logistic regression for MI incidence at 30, 90, and 365 days postoperatively, and multiple Cox proportional hazards models for MI-related mortality.

**Results:**

In total, 1,148,298 procedures were included in the final analysis. Of those undergoing hip arthroplasty, 713 (0.13%) patients experienced a MI within 30 days postoperatively, rising to 947 (0.17%) at 90 days and 1,989 (0.38%) patients at one year. In the knee arthroplasty cohort, the number of patients experiencing a MI was 656 (0.11%) at 30 days, 890 (0.15%) at 90 days, and 1,853 (0.32%) at one year postoperatively. Factors associated with increased MI incidence included age, male sex, non-white ethnicities, geographical region, higher deprivation status, and elevated comorbidity scores. Weighted logistic regression demonstrated significantly lower odds of MI, and MI-related mortality in patients undergoing UKA and HRA compared with TKA and THA, respectively.

**Conclusion:**

In England, post-arthroplasty MI remains uncommon but carries significant mortality risk. Bone-preserving procedures are associated with a significantly reduced incidence of MI and subsequent mortality. These findings emphasize the importance of careful patient and treatment selection, and targeted cardiovascular risk mitigation, to optimize postarthroplasty outcomes.

Cite this article: *Bone Jt Open* 2026;7(8):1046–1055.

## Introduction

Hip and knee arthroplasty rank among the most common elective procedures worldwide, with over 200,000 annual procedures in the UK alone.^1,2^ Much of this demand is driven by osteoarthritis (OA), which affects 7% of the population and is a leading cause of hip and knee disability, often necessitating arthroplasty in end-stage disease.^3,4^ In the UK and elsewhere, an ageing population has fuelled rising demand, which is expected to grow by up to 40% by 2060.^3^ These procedures can deliver transformative outcomes, with substantial improvements in pain, functional status, and ultimately quality of life.^5^

Despite its well-established benefits, lower-limb joint arthroplasty is not without complications, which may include infection, thromboembolism, periprosthetic fractures, peripheral nerve damage, and joint dislocation, particularly in patients with higher BMIs.^6-8^ Another important postoperative complication associated with hip and knee arthroplasty is myocardial infarction (MI), which is significant as one of the leading causes of mortality following lower-limb joint arthroplasty.^9-11^ A retrospective cohort study by Pedersen et al^12^ reported a MI incidence of 0.5% following total hip arthroplasty (THA) and total knee arthroplasty (TKA) at 90 days postoperatively.

To date, several studies have investigated the incidence of MI following lower-limb joint arthroplasty.^11-14^ However, data within the context of the UK remain limited. Moreover, most studies exclusively focused on unicompartmental knee arthroplasty (UKA) or hip resurfacing arthroplasty (HRA) and restricted their analyses to short-term outcomes (i.e. 30 to 90 days). This study aimed to assess the long-term incidence of MI following different types of hip and knee arthroplasty and subsequent mortality in a UK-based cohort.

## Methods

### Study design

We conducted a retrospective cohort study using the UK Clinical Practice Research Datalink (CPRD) linked to Hospital Episode Statistics (HES) and the Office for National Statistics (ONS) mortality registry. We identified adults undergoing primary THA, HRA, TKA, or UKA between January 1998 and March 2021 using Office of Population Censuses and Surveys-4 (OPCS-4) procedure codes from HES (Supplementary Table 1). Records were excluded if they had missing referral dates, invalid or missing operation dates, implausible admission-discharge sequences, age < 18 years, missing Index of Multiple Deprivation (IMD) values, or death dates preceding the recorded operation date. Operations involving both hip and knee procedures on the same date were also excluded. Only cases with valid event dates within the study period were retained.

### Data linkage

All demographic and mortality data were linked to HES records using a unique patient identifier. Within HES, individual care episodes and hospital stays were tracked using this identifier in combination with the spell (admission) number and/or the unique episode key. Mortality data came from the ONS, providing validated records on cause and date of death. A complete list of datasets and their date ranges is provided in Supplementary Table 2. Additional demographic variables, including BMI and comorbidities, were sourced from general practice records in the CPRD AURUM dataset.

### Participants

All included patients were aged 18 years or older and underwent at least one primary TKA, THA, UKA, or HRA in England between 2 January 1998 and 29 March 2021. Patients were stratified into two procedural cohorts: TKA compared with UKA, and THA compared with HRA, to facilitate comparative analyses of the odds of MI between conventional and less invasive surgical approaches.^15-18^

The baseline patient demographic details for the hip arthroplasty cohort are in Table I.

**Table I.** Patient demographic details in the hip arthroplasty cohort.

Variable	30 days postop			90 days postop			1 year postop		p-value
	No MI (n = 549,160)	MI (n = 713)	p-value	No MI (n = 545,284)	MI (n = 947)	p-value	No MI (n = 527,473)	MI (n = 1,989)	
**Mean age at operation, yrs (SD)**	68.1 (12.3)	77.6 (9.2)	< 0.001	68 (12)	77 (9)	< 0.001	68 (12)	72 (15)	< 0.001*
**Sex, n (%)**			< 0.001			< 0.001			< 0.001†
Female	338,918 (62)	356 (50)		336,710 (62)	479 (51)		326,032 (62)	1,088 (55)	
Male	210,242 (38)	357 (50)		208,574 (38)	468 (49)		201,441 (38)	901 (45)	
**Ethnicity, n (%)**			0.098			0.232			< 0.001†
White	442,254 (81)	594 (83)		439,309 (81)	783 (83)		425,377 (81)	1,566 (79)	
Not white	11,746 (2.1)	17 (2.4)		11,658 (2.1)	20 (2.1)		11,110 (2.1)	135 (6.8)	
Unknown	95,160 (17)	102 (14)		94,317 (17)	144 (15)		90,986 (17)	288 (14)	
**Region, n (%)**			0.028			0.030			< 0.001†
East Midlands	13,093 (2.4)	23 (3.2)		13,005 (2.4)	32 (3.4)		12,610 (2.4)	59 (3.0)	
East of England	26,298 (4.8)	35 (4.9)		26,121 (4.8)	49 (5.2)		25,372 (4.8)	90 (4.5)	
London	63,629 (12)	85 (12)		63,204 (12)	112 (12)		60,595 (11)	213 (11)	
North East	17,352 (3.2)	32 (4.5)		17,181 (3.2)	35 (3.7)		16,576 (3.1)	68 (3.4)	
North West	95,534 (17)	149 (21)		94,753 (17)	196 (21)		91,925 (17)	361 (18)	
South East	129,520 (24)	157 (22)		128,712 (24)	204 (22)		124,239 (24)	380 (19)	
South West	84,404 (15)	88 (12)		83,750 (15)	124 (13)		81,384 (15)	254 (13)	
West Midlands	98,084 (18)	121 (17)		97,492 (18)	164 (17)		94,353 (18)	488 (25)	
Yorkshire and the Humber	21,246 (3.9)	23 (3.2)		21,066 (3.9)	31 (3.3)		20,419 (3.9)	76 (3.8)	
**IMD 2019, n (%)**			0.025			0.018			< 0.001†
1 (most deprived)	64,702 (12)	76 (11)		64,289 (12)	102 (11)		62,146 (12)	195 (9.8)	
2	62,293 (11)	89 (12)		61,851 (11)	116 (12)		59,869 (11)	218 (11)	
3	64,046 (12)	75 (11)		63,611 (12)	101 (11)		61,599 (12)	191 (9.6)	
4	65,213 (12)	95 (13)		64,738 (12)	117 (12)		62,665 (12)	234 (12)	
5	56,677 (10)	53 (7.4)		56,274 (10)	74 (7.8)		54,460 (10)	161 (8.1)	
6	57,698 (11)	71 (10.0)		57,276 (11)	102 (11)		55,444 (11)	217 (11)	
7	53,302 (9.7)	65 (9.1)		52,901 (9.7)	81 (8.6)		51,183 (9.7)	168 (8.4)	
8	43,835 (8.0)	53 (7.4)		43,502 (8.0)	77 (8.1)		42,030 (8.0)	151 (7.6)	
9	42,819 (7.8)	69 (9.7)		42,532 (7.8)	88 (9.3)		41,123 (7.8)	203 (10)	
10 (least deprived)	38,575 (7.0)	67 (9.4)		38,310 (7.0)	89 (9.4)		36,954 (7.0)	251 (13)	
**Preop 5-year CCI, n (%)**			< 0.001			< 0.001			< 0.001†
0	446,268 (81)	471 (66)		443,623 (81)	624 (66)		431,421 (82)	1,198 (60)	
1	71,665 (13)	114 (16)		71,105 (13)	160 (17)		68,231 (13)	298 (15)	
2	19,578 (3.6)	82 (12)		19,194 (3.5)	107 (11)		17,895 (3.4)	161 (8.1)	
3+	11,649 (2.1)	46 (6.5)		11,362 (2.1)	56 (5.9)		9,926 (1.9)	332 (17)	
**Mean BMI, kg/m** ^**2**^ **(SD)**	27.9 (5.2)	27.3 (4.8)	0.128	27.9 (5.3)	27.4 (5.0)	0.107	27.9 (5.3)	27.7 (5.2)	0.410*
Missing	318,348	404		316,310	540		306,661	1,218	
**Surgery type, n (%)**			< 0.001			< 0.001			< 0.001†
HRA	14,135 (2.6)	4 (0.6)		14,117 (2.6)	4 (0.4)		13,976 (2.6)	13 (0.7)	
THA	535,025 (97)	709 (99)		531,167 (97)	943 (100)		513,497 (97)	1,976 (99)	
**Mean follow-up, mths (SD)**	100 (66)	66 (56)	< 0.001	100 (64)	68 (58)	< 0.001	103 (63)	72 (59)	< 0.001*

All percentages were reported to two significant figures, with percentages below 10% presented to one decimal place.

* Mann-Whitney U test.

† Pearson’s chi-squared test.

CCI, Charlson Comorbidity Index; HRA, hip resurfacing arthroplasty; IMD, Index of Multiple Deprivation; MI, myocardial infarction; THA, total hip arthroplasty.

The baseline patient demographic details for the knee arthroplasty cohort can be seen in Table II.

**Table II.** Patient demographic details in the knee arthroplasty cohort.

Variable	30 days postop			90 days postop			1 year postop		p-value
	No MI (n 592,508)	MI (n = 656)	p-value	No MI (n = 589,948)	MI (n = 890)	p-value	No MI (n = 575,593)	MI (n = 1,853)	
**Mean age at operation, yrs (SD)**	69 (10)	77 (8)	< 0.001	69 (10)	76 (8)	< 0.001	69 (10)	72 (13)	< 0.001*
**Sex, n (%)**			< 0.001			< 0.001			< 0.001†
Female	350,201 (59)	267 (41)		348,894 (59)	366 (41)		341,033 (59)	820 (44)	
Male	242,307 (41)	389 (59)		241,054 (41)	524 (59)		234,560 (41)	1,033 (56)	
**Ethnicity, n (%)**			< 0.001			0.028			0.019†
Not white	35,890 (6.1)	61 (9.3)		35,807 (6.1)	69 (7.8)		34,790 (6.0)	112 (6.0)	
Unknown	93,618 (16)	86 (13)		93,022 (16)	120 (13)		90,339 (16)	247 (13)	
White	463,000 (78)	509 (78)		461,119 (78)	701 (79)		450,464 (78)	1,494 (81)	
**Region, n (%)**			< 0.001			< 0.001			< 0.001†
East Midlands	14,714 (2.5)	28 (4.3)		14,648 (2.5)	36 (4.0)		14,344 (2.5)	56 (3.0)	
East of England	26,475 (4.5)	24 (3.7)		26,395 (4.5)	31 (3.5)		25,786 (4.5)	62 (3.3)	
London	81,848 (14)	112 (17)		81,579 (14)	146 (16)		79,098 (14)	277 (15)	
North East	21,409 (3.6)	19 (2.9)		21,285 (3.6)	25 (2.8)		20,773 (3.6)	71 (3.8)	
North West	102,810 (17)	146 (22)		102,244 (17)	196 (22)		99,983 (17)	354 (19)	
South East	134,410 (23)	125 (19)		133,933 (23)	165 (19)		130,399 (23)	344 (19)	
South West	80,731 (14)	72 (11)		80,268 (14)	116 (13)		78,639 (14)	234 (13)	
West Midlands	106,893 (18)	112 (17)		106,499 (18)	148 (17)		103,875 (18)	398 (21)	
Yorkshire and the Humber	23,218 (3.9)	18 (2.7)		23,097 (3.9)	27 (3.0)		22,696 (3.9)	57 (3.1)	
**IMD 2019, n (%)**			0.364			0.335			0.007†
1	63,506 (11)	77 (12)		63,227 (11)	98 (11)		61,580 (11)	191 (10)	
2	62,821 (11)	75 (11)		62,584 (11)	93 (10)		60,981 (11)	196 (11)	
3	65,878 (11)	72 (11)		65,567 (11)	91 (10)		63,950 (11)	192 (10)	
4	68,052 (11)	73 (11)		67,752 (11)	97 (11)		66,091 (11)	219 (12)	
5	59,745 (10)	80 (12)		59,491 (10)	101 (11)		58,062 (10)	187 (10)	
6	62,971 (11)	53 (8.1)		62,706 (11)	76 (8.5)		61,214 (11)	170 (9.2)	
7	59,267 (10)	59 (9.0)		58,999 (10)	85 (9.6)		57,596 (10)	177 (9.6)	
8	52,259 (8.8)	59 (9.0)		52,028 (8.8)	92 (10)		50,815 (8.8)	164 (8.9)	
9	51,224 (8.6)	51 (7.8)		51,006 (8.6)	76 (8.5)		49,803 (8.7)	160 (8.6)	
10	46,785 (7.9)	57 (8.7)		46,588 (7.9)	81 (9.1)		45,501 (7.9)	197 (11)	
**Preop 5-year CCI, n (%)**			< 0.001			< 0.001			< 0.001†
0	469,501 (79)	456 (70)		467,591 (79)	609 (68)		457,497 (79)	1,192 (64)	
1	90,988 (15)	110 (17)		90,580 (15)	159 (18)		88,048 (15)	337 (18)	
2	20,712 (3.5)	55 (8.4)		20,563 (3.5)	70 (7.9)		19,733 (3.4)	117 (6.3)	
3+	11,307 (1.9)	35 (5.3)		11,214 (1.9)	52 (5.8)		10,315 (1.8)	207 (11)	
**Mean BMI, kg/m^2^ (SD)**	30.1 (5.5)	28.4 (4.8)	< 0.001	30.1 (5.5)	28.8 (5.3)	< 0.001	30.1 (5.5)	29.2 (5.2)	< 0.001*
Unknown	347,683	371		346,265	502		338,370	1,064	
**Surgery type, n (%)**			< 0.001			< 0.001			< 0.001†
TKA	537,660 (91)	641 (98)		535,445 (91)	860 (97)		522,896 (91)	1,764 (95)	
UKA	54,848 (9.3)	15 (2.3)		54,503 (9.2)	30 (3.4)		52,697 (9.2)	89 (4.8)	
**Mean follow-up, mths (SD)**	98 (61)	78 (58)	< 0.001	98 (60)	77 (58)	< 0.001	100 (59)	82 (60)	< 0.001*

All percentages were reported to two significant figures, with percentages below 10% presented to one decimal place.

* Mann-Whitney U test.

† Pearson’s chi-squared test.

CCI, Charlson Comorbidity Index; IMD, Index of Multiple Deprivation; TKA, total knee arthroplasty; UKA, unicompartmental knee arthroplasty.

### Data cleaning

Patients with a recorded sex other than male or female were excluded. Records were also removed if they included a date of death before the index procedure. Additional exclusions applied to records missing core demographic information, such as age, sex, date of operation, admission, discharge, or IMD 2019 score. Records with inconsistent episode timing, such as admission after discharge or episode start after episode end, were also excluded.

### Outcomes and exposure

The primary outcome was myocardial infarction at 30 days, 90 days, and one year following surgery. Analyses were stratified by surgical procedure, with comparisons conducted for THA versus HRA and for TKA versus UKA. The main exposure of interest was the type of surgery received. Myocardial infarction was defined using International Classification of Diseases of the World Health Organization,^19^ tenth revision (ICD-10) codes I21 and I22.^20^ A secondary outcome included overall MI-related mortality across the entire follow-up period, as defined in the HES dataset.

### Statistical analysis

Categorical variables were compared across groups using chi-squared tests. Continuous variables were compared using Mann-Whitney U tests, and summarized as mean (SD). All analyses were conducted at the level of the surgical operation. No adjustment for clustering of patients within surgeon or hospital was made. In our experience, this does not affect the results and such models are harder to make converge. Comorbidities were quantified using the Charlson Comorbidity Index (CCI).^21,22^ Scores were summed to produce an overall comorbidity burden, which was categorized in this study as none (0), mild (1), moderate (2), or severe (≥ 3) for analytical purposes.^15^

To minimize confounding differences between participants of each operation we used an inverse probability weighting approach. We calculated the probability of a patient undergoing HRA compared with THA, and UKA compared wtih TKA respectively, for each individual with an individual-level propensity score based on age at operation, sex, IMD 2019, CCI score, ethnicity, and region. The propensity scores were then used to assign a weight to each individual in the total joint replacement group that was inversely proportion to the probability of their being a participant in the HRA or UKA group, for knees and hips separately.

A weighted logistic regression was then employed to identify factors associated with MI at the defined timepoints. All models reported estimates with 95% CIs and associated p-values. Censoring was applied, and MI incidence was only recorded for patients with complete follow-up for the specified time window. Otherwise, values were set to missing (N/A). THA and TKA were used as the reference groups in their respective comparisons, with odds ratios (ORs) or coefficients greater than 1 indicating greater odds or higher values among HRA or UKA, respectively.

Cox proportional hazards models estimated hazard ratios (HRs) were calculated for MI-related death during follow-up. Both unadjusted and adjusted models were fitted. The adjusted models included age at operation, sex, ethnicity, region, IMD decile, and CCI score. The proportional hazards assumption was formally tested and met, including the primary exposure of procedure type. Survival analysis was undertaken for hips and knees separately, similar to the MI incidence analysis.

All analyses were carried out in R (v. 4.4.1; R Foundation for Statistical Computing, Austria). Statistical significance was defined as p < 0.05. This study and CPRD data access was approved by the Medicines and Healthcare products Regulatory Agency Independent Scientific Advisory Committee (reference ID: 22_002170).

## Results

Of the 1,182,211 inputs in the dataset, 1,148,298 procedures met the inclusion criteria and were included in the final analysis. Among these, 535,734 were THA, 538,301 TKA, 14,139 HRA, and 54,863 UKA.

### Hip arthroplasty

At 30 days after surgery, 713 patients (0.13%) had experienced a MI. This figure rose to 947 (0.17%) and 1,989 (0.38%) at 90 days and one year postoperatively, respectively. The mean age of patients who experienced a MI within 30 days postoperatively was significantly higher those who did not ( p < 0.001, Mann-Whitney U test). Moreover, a higher proportion of male patients suffered from postoperative MI compared with their female counterparts at all three follow-up timepoints. Preoperative CCI scores were significantly lower in those who did not experience a MI. Both geographical region and IMD status demonstrated significant differences between the MI and non-MI cohort across all follow-up timepoints (Table I).

### Knee arthroplasty

A total of 656 patients (0.11%) had suffered from a MI within the 30-day postoperative period, which increased cumulatively to 890 patients (0.15%) at 90 days and 1,853 patients (0.32%) at one-year follow-up. Similarly to the hip arthroplasty patient cohort, those who experienced a MI were significantly older, more commonly male, and had significantly higher preoperative CCI scores, compared with the non-MI cohort. Ethnicity differed significantly across all follow-up timepoints, while IMD status only demonstrated a significant difference at the final one-year follow-up (Table II).

### Logistic regression analysis – hip arthroplasty

As seen in Table III, the unadjusted odds of MI at 30 days postoperatively was significantly lower in those undergoing HRA compared with THA (OR 0.21, 95% CI 0.07 to 0.50; p = 0.002), and this difference persisted at the 90-day and one-year postoperative timepoints. In contrast, weighted logistic regression detected a significant difference in odds between the two procedures only at the one-year postoperative interval, with HRA associated with significantly reduced odds (OR 0.37, 95% CI 0.19 to 0.68; p < 0.001) at this timepoint (Table III).

**Table III.** Unadjusted and weighted logistic regression model (inverse probability weighting) of myocardial infarction (MI) incidence comparing total hip arthroplasty (THA) and hip resurfacing arthroplasty (HRA).

Variable	30-day MI			90-day MI			1-year MI		
	OR	95% CI	p-value	OR	95% CI	p-value	OR	95% CI	p-value
**Unadjusted**									
THA	N/A	N/A		N/A	N/A		N/A	N/A	
HRA	0.21	0.07 to 0.50	0.002	0.16	0.05 to 0.37	< 0.001	0.24	0.13 to 0.40	< 0.001
**Adjusted**									
THA	N/A	N/A		N/A	N/A		N/A	N/A	
HRA	0.63	0.16 to 2.11	0.463	0.43	0.12 to 1.29	0.154	0.37	0.19 to 0.68	0.002

N/A, not applicable; OR, odds ratio.

### Logistic regression analysis – knee arthroplasty

Unadjusted and weighted logistic regression analysis revealed that UKA was associated with significantly reduced odds of myocardial infarction at all three postoperative timepoints (Table IV).

**Table IV.** Unadjusted and weighted logistic regression model (inverse probability weighting) of myocardial infarction (MI) incidence comparing total knee arthroplasty (TKA) and unicompartmental knee arthroplasty (UKA).

Variable	30-day MI			90-day MI			1-year MI		
	OR	95% CI	p-value	OR	95% CI	p-value	OR	95% CI	p-value
**Unadjusted**									
TKA	N/A	N/A		N/A	N/A		N/A	N/A	
UKA	0.23	0.13 to 0.37	< 0.001	0.34	0.23 to 0.48	< 0.001	0.50	0.40 to 0.62	< 0.001
**Adjusted**									
TKA	N/A	N/A		N/A	N/A		N/A	N/A	
UKA	0.38	0.20 to 0.67	0.001	0.53	0.34 to 0.82	0.005	0.53	0.41 to 0.68	< 0.001

OR, odds ratio.

### Cox hazard regression survival analysis – hip arthroplasty

In total, there were 5,973 (1.1%) MI-related deaths in the hip arthroplasty cohort. Unadjusted Cox regression analysis revealed that HRA was significantly associated with a reduced risk of MI-related mortality compared with THA (HR 0.24, 95% CI 0.19 to 0.30; p < 0.001) (Table V). Multiple Cox regression also revealed a significantly lower hazard of death secondary to a MI in patients undergoing HRA, albeit to a lower extent (HR 0.71, 95% CI 0.56 to 0.92; p = 0.009). Additionally, age, male sex, unknown ethnicity, and a preoperative CCI score of 1 or greater were all independently associated with a risk of postoperative MI-related mortality (Table V).

**Table V.** Unadjusted and multiple Cox hazard regression model of survival comparing total hip arthroplasty (THA) and hip resurfacing arthroplasty (HRA).

Variable	HR	95% CI	p-value
**Unadjusted**			
THA	N/A	N/A	
HRA	0.24	0.19 to 0.30	< 0.001
**Adjusted**			
THA	N/A	N/A	
HRA	0.71	0.56 to 0.92	0.009
**Age at operation, yrs**	1.09	1.08 to 1.09	< 0.001
**Sex**			
Female	N/A	N/A	
Male	2.04	1.93 to 2.14	< 0.001
**Ethnicity**			
White	N/A	N/A	
Not white	0.91	0.72 to 1.15	0.431
Unknown	1.10	1.03 to 1.17	0.003
**Preop 5-yr CCI**			
0	N/A	N/A	
1	1.56	1.45 to 1.68	< 0.001
2	2.28	2.03 to 2.55	< 0.001
3+	3.31	2.88 to 3.81	< 0.001

CCI, Charlson Comorbidity Index; HR, hazard ratio.

### Cox hazard regression survival analysis – knee arthroplasty

In total, there were 6,450 (1.1%) MI-related deaths in the knee arthroplasty cohort. UKA was associated with significantly reduced risk of postoperative mortality secondary to a MI compared with TKA, according to both unadjusted (HR 0.50, 95% CI 0.44 to 0.57; p < 0.001) and multiple Cox regression (HR 0.71, 95% CI 0.62 to 0.80, p < 0.001) analysis. Furthermore, age, male sex, and a preoperative CCI score of 1 or greater were also independently associated with heightened hazards of MI-related death following knee arthroplasty (Table VI).

**Table VI.** Unadjusted and multiple Cox hazard regression model of survival comparing total knee arthroplasty (TKA) and unicompartmenal knee arthroplasty (UKA).

Variable	HR	95% CI	p-value
**Unadjusted**			
TKA	N/A	N/A	
UKA	0.50	0.44 to 0.57	< 0.001
**Adjusted**			
TKA	N/A	N/A	
UKA	0.71	0.62 to 0.80	< 0.001
**Age at operation, yrs**	1.09	1.08 to 1.09	< 0.001
**Sex**			
Female	N/A	N/A	
Male	2.05	1.95 to 2.15	< 0.001
**Ethnicity**			
White	N/A	N/A	
Not white	1.09	0.98 to 1.22	0.121
Unknown	1.03	0.97 to 1.10	0.296
**Preop 5-yr CCI**			
0	N/A	N/A	
1	1.53	1.43 to 1.63	< 0.001
2	2.07	1.84 to 2.32	< 0.001
3+	2.39	2.03 to 2.82	< 0.001

CCI, Charlson Comorbidity Index; HR, hazard ratio.

## Discussion

To our knowledge, this is the first study to directly investigate and compare the incidence of MI and subsequent mortality across different lower-limb arthroplasty procedures in the UK. Our study highlights several useful findings, most notably that incidence of a MI following hip arthroplasty was 0.13% at 30 days, rising to 0.18% at 90 days and 0.38% at one year postoperatively. The incidence was comparable in patients undergoing knee arthroplasty (0.11% at 30 days, 0.15% at 90 days, and 0.32% at one year). Factors associated with increased MI incidence included age, male sex, non-white ethnicities, geographical region, higher deprivation status, and comorbidity. Additionally, when analyzing each procedure individually, UKA and HRA were associated with a reduced risk of postoperative MI and a nearly 30% reduction in mortality risk compared with traditional joint arthroplasties.

Several aspects of our research distinguish it from prior work. First, with a sample size of over one million patients, our study leverages the largest dataset of arthroplasty patients in the UK to date. In this context, it offers a more diverse and generalizable patient population compared with previous research, in turn enabling an accurate reflection of the current reality. Second, it utilizes long-term follow-up data, extending up to one year for MI rates and some 20 years postoperatively for mortality. Third, our inclusion of UKA and HRA procedures provides unique insights into the impact of procedure type on MI risk.

Our findings align with the existing literature regarding the incidence of MI following lower-limb joint arthroplasty. A retrospective cohort study by Lu et al^14^ comprising 19,912 UK-based patients, found that 308 patients (2.2%) undergoing TKA and 128 patients undergoing THA (2.1%) developed MI postoperatively over a 12-year study period. While the incidence reported by Lu et al^14^ was substantially higher than in our study, this may partly reflect their cohort being restricted to TKA and THA, whereas our analysis also included UKA and HRA. In this context, the generalizability of their findings may be limited. Additionally, Lu et al^14^ did not examine mortality following postoperative MI, highlighting another key strength of our study. A study by Pedersen et al^12^ reported a 30-day MI incidence of 0.4% following THA, which increased to 0.5% at 60 days and 0.6% at 90 days postoperatively. While broadly comparable, their study did not adjust for key confounders, which limits the validity of their findings. In contrast, our study employed inverse probability weighting at a 5:1 ratio, accounting for various clinical and demographic variables, including age, sex, and ethnicity, ensuring robustness in our analysis.

Evidence comparing cardiovascular outcomes between procedure types is more limited, but broadly consistent with our findings. Liddle et al^23^ conducted a large retrospective cohort study using the National Joint Registry, in which a total of over 100,000 TKA and UKA patients were propensity score matched, and reported significantly lower odds of MI following UKA compared with TKA after adjusting for demographic details (OR 0.53, 95% CI 0.31 to 0.90; p = 0.018). Comparative data for HRA and THA are more sparse. Darrith et al^24^ reported outcomes from a single-surgeon cohort comparing hip and knee arthroplasty procedures that were originally analyzed according to inpatient and outpatient setting. For hip arthroplasty, 70 HRAs were compared with 178 THAs. To allow consistency with our analyses, complication rates were pooled across care settings for HRA and THA only, demonstrating similar 90-day rates of major complications, defined as a composite of death, MI, and other serious adverse events (4.1% for THA vs 1.4% for HRA). No formal inter-procedural statistical comparison was performed following pooling, reflecting the structure of the published data, although the original analyses included risk adjustment within care-setting strata. In keeping with these observations, our adjusted analyses demonstrate lower MI incidence and MI-related mortality following UKA and HRA compared with their more invasive counterparts, suggesting that bone-preserving procedures may be associated with lower cardiovascular risk in appropriately selected patients beyond baseline risk alone. Further longitudinal studies are required to confirm these hypotheses.

Beyond procedure type, increased odds of postoperative MI and MI-related mortality were associated with older age, male sex, higher comorbidity burden, greater socioeconomic deprivation, and regional variation. These associations are consistent with established cardiovascular risk literature, which links age, male sex, obesity, and comorbidity with increased perioperative and longer-term MI risk.^25,26^ These findings reinforce the importance of preoperative cardiovascular risk assessment and optimization in patients undergoing lower-limb arthroplasty.

Comorbidities were assessed using the CCI, and while recalculated preoperatively per procedure, may not fully capture frailty or cumulative clinical burden.^27^ Additionally, the absence of data on relevant medication use, such as cardiac agents, as well as lifestyle factors, including physical activity and smoking habits, may have influenced the observed outcomes. The specific clinical causes of MI events were not available, so we are unable to be certain that these were directly attributable to the arthroplasty procedure, or secondary to an unrelated cause. Despite statistical adjustment, residual confounding likely remains, particularly for factors that may not be available in observational data, such as physiological reserve, preoperative activity levels, and sub-clinical cardiovascular disease. In addition, although UKA and HRA may offer clinical benefit, they are generally reserved for more carefully selected patients with less advanced disease and lower frailty, which may partly explain their lower observed cardiovascular risk compared with TKA and THA.^28,29^ This potential selection bias may limit attribution of the observed findings to true procedural benefit.

These findings may help to inform preoperative counselling by supporting individualized discussions of cardiovascular risk before arthroplasty, particularly in older patients, those with greater comorbidity burden, and those being considered for more limited bone-preserving procedures. However, the reduced cardiovascular risk observed with UKA and HRA should be interpreted cautiously. Preoperative counselling should therefore emphasize careful risk stratification, optimization of modifiable cardiovascular factors, and shared decision-making based on overall patient fitness alongside procedure type.

In conclusion, this national-level cohort study revealed that MI following lower-limb joint arthroplasty was uncommon, but associated with substantial mortality risk. Importantly, UKA and HRA were significantly associated with lower odds of postoperative MI and subsequent mortality, even after adjustment for confounders, when compared with their conventional counterparts, likely reflecting the less invasive nature of these procedures. These findings emphasize the importance of careful patient and treatment selection, and targeted cardiovascular risk mitigation to optimize postarthroplasty outcomes. The findings can also be used to assist in patient-centred operative decision-making.

**Take home message**

- This national-level cohort study revealed that myocardial infarction (MI) following lower-limb joint arthroplasty was uncommon, but associated with substantial mortality risk.

- Importantly, unicompartmental knee arthroplasty and hip resurfacing arthroplasty were significantly associated with lower odds of postoperative MI and subsequent mortality compared to total knee arthroplasty and total hip arthroplasty, respectively.

- These findings emphasize the importance of careful patient and treatment selection, and targeted cardiovascular risk mitigation to optimize post-arthroplasty outcomes.

## Data Availability

The datasets generated and analyzed in the current study are not publicly available due to data protection regulations. Access to data is limited to the researchers who have obtained permission for data processing. Further inquiries can be made to the corresponding author.
